# Multidisciplinary Taiwan consensus for the use of conventional TACE in hepatocellular carcinoma treatment

**DOI:** 10.3389/fonc.2023.1186674

**Published:** 2023-06-20

**Authors:** Pi-Yi Chang, Rheun-Chuan Lee, Po-Chin Liang, Yi-Sheng Liu, Vicent P. Chuang, Ding-Kwo Wu, Yu-Fan Cheng, Jen-I. Huang, Hsiuo-Shan Tseng, Chien-Fu Hung, Reng-Hong Wu, Ming-Chih Chern, Hua-Ming Cheng, Chih-Horng Wu, She-Meng Cheng, Chia-Ling Chiang, Huei-Lung Liang

**Affiliations:** ^1^ Department of Radiology, Taichung Veterans General Hospital, Taichung, Taiwan; ^2^ Department of Radiology, Taipei Veterans General Hospital, Taipei, Taiwan; ^3^ Department of Radiology, National Taiwan University Hospital Hsin-Chu Branch, Hsinchu, Taiwan; ^4^ Department of Medical Imagine, National Cheng Kung University Hospital, Tainan, Taiwan; ^5^ Department of Radiology, Koo Foundation Sun Yat-Sen Cancer Center, Taipei, Taiwan; ^6^ Department of Radiology, Kaohsiung Medical University Hospital, Kaohsiung, Taiwan; ^7^ Department of Radiology, Kaohsiung Chang Gung Memorial Hospital, Kaohsiung, Taiwan; ^8^ Department of Radiology, Tungs’ Taichung Metroharbor Hospital, Taichung, Taiwan; ^9^ Department of Radiology, Cheng Hsin General Hospital, Taipei, Taiwan; ^10^ Department of Radiology, Chang−Gung Memorial Hospital, Taoyuan, Taiwan; ^11^ Department of Radiology, Chi Mei Medical Center, Tainan, Taiwan; ^12^ Department of Radiology, MacKay Memorial Hospital, Taipei, Taiwan; ^13^ Department of Radiology, Kaohsiung Veterans General Hospital, Kaohsiung, Taiwan

**Keywords:** chemoembolization, cTACE, lipiodol, hepatocellular carcinoma, consensus

## Abstract

Developed in early 1980s, transarterial chemoembolization (TACE) with Lipiodol was adopted globally after large-scale randomized control trials and meta-analyses proving its effectiveness were completed. Also known as “conventional TACE” (cTACE), TACE is currently the first-line treatment for patients with unresectable intermediate stage hepatocellular carcinoma (HCC) and delivers both ischemic and cytotoxic effects to targeted tumors. Although new technology and clinical studies have contributed to a more comprehensive understanding of when and how to apply this widely-adopted therapeutic modality, some of these new findings and techniques have yet to be incorporated into a guideline appropriate for Taiwan. In addition, differences in the underlying liver pathologies and treatment practices for transcatheter embolization between Taiwan and other Asian or Western populations have not been adequately addressed, with significant variations in the cTACE protocols adopted in different parts of the world. These mainly revolve around the amount and type of chemotherapeutic agents used, the type of embolic materials, reliance on Lipiodol, and the degree of selectiveness in catheter positioning. Subsequently, interpreting and comparing results obtained from different centers in a systematic fashion remain difficult, even for experienced practitioners. To address these concerns, we convened a panel of experts specializing in different aspects of HCC treatment to devise modernized recommendations that reflect recent clinical experiences, as well as cTACE protocols which are tailored for use in Taiwan. The conclusions of this expert panel are described herein.

## 1 Introduction

Hepatocellular carcinoma (HCC) is the predominant form of liver cancer in many countries and is the third most common cause of cancer-related death in the Asia-Pacific region. Chronic hepatitis B virus (HBV) and hepatitis C virus (HCV) infections are the two chief contributors to cases of HCC in Asia, with 75% of the more than 350 million chronic HBV carriers in the world living in the Asia-Pacific region (1, 2). Since the virus is endemic to much of the Asia-Pacific region, and with vertical transmission being common, chronic HBV infection remains by far the largest single factor for developing liver cancer, accounting for more than 70% of new cases diagnosed annually (3, 4). In contrast to the rest of Asia, HCV-related infection is responsible for 80% to 90% of all HCC cases in Japan. However, over the past several decades, the prevalence of HCV has risen in many Asia-Pacific countries, with prominent increases in HCV-related HCC being found in Taiwan (5). In Taiwan, HCC is the second leading cause of cancer-related death, and the majority of cases are still associated with chronic HBV infection (6).

While surgical resection continues to be the mainstream treatment in well-selected patients, less-invasive techniques such as conventional chemoembolization (cTACE) have become globally accepted as an effective treatment for patients with unresectable HCC (7, 8). Previous studies have shown that HCC staging based on the tumor-node-metastasis (TNM) system and therapy selection *via* the Barcelona clinic liver cancer (BCLC) staging system are applicable for the Taiwanese population and demonstrate clear survival benefits (9–13). Initially developed in Japan in the early 1980s, cTACE has been established worldwide as the standard treatment for intermediate-stage HCC without portal vein invasion (14–17). At its core, cTACE involves the use of specialized catheters to localize arterial administration of one or more cytotoxic drugs to hepatic tumors. Common therapeutic agents include doxorubicin (18) and cisplatin (19), which are thoroughly mixed with ethiodized oil (Lipiodol, Guerbet, France) prior to injection. This may then be followed by embolization of tumor-feeding vessels with particulate embolic agents, such as gelatin sponge or polyvinyl alcohol (PVA) particles (16). cTACE operates on the principle of delivering high local concentrations of a chemotherapeutic drug into liver tumors *via* the hepatic arterial supply, followed by the embolization of these arteries with various types of particles in order to reduce arterial blood flow. The surrounding liver parenchyma is spared due to its preferential blood supply by the portal venous circulation. Consequently, systemic toxicity can be minimized, as most of the injected chemotherapeutic agent remains within the tumor bed rather than escaping into systemic circulation. Recently, Lencioni (2016) reported the results of a meta-analysis on the efficacy of cTACE, which included a total of 10,108 HCC patients treated by Lipiodol cTACE. The systematic review showed a median overall survival (OS) of 19.4 months and a 5-year OS of 32.4% (20). A survival analysis (2018) which compared OS between TACE with a tyrosine kinase inhibitor (orantinib) or cTACE with a placebo found that patients without portal vein invasion (PVI) reached a median OS of 32.3 months using cTACE alone, which was not significantly worse than cTACE with a target therapy (21).

Over the past 30 years, numerous studies have examined the application of cTACE in HCC treatment, with more standardized concepts in patient selection and treatment protocols evolving as interventional radiologists around the world developed a more comprehensive understanding of this therapeutic modality. However, although cTACE has been performed in Taiwan for more than 30 years and interventional techniques and devices have matured in recent decades, a set of standardized best practices (the specifics of preparing the Lipiodol and drug emulsion, its method of administration, as well as the embolic agents used) needs to be established in order to be widely integrated into a modern model for oncologic treatment of liver tumors.

To that end, a group of expert physicians was convened on September 9, 2018, to develop an up-to-date technical guideline on the use of cTACE for HCC treatment in Taiwan, based on their accumulated clinical experience and research data. The experts in this panel all had extensive experience in the field of interventional radiology, and their opinions reflect the best standards of practice at leading medical centers for HCC treatment in Taiwan. During the preparation of this manuscript, a consensus statement on the treatment of intermediate-stage HCC was established in 2019 during the 10th Annual Meeting of the Asia-Pacific Primary Liver Cancer Expert (APPLE), which was held in Sapporo, Japan (22). A virtual meeting of the panel members was later convened in October of 2021 to refine the statement of cTACE ineligibility. The following statements summarize the consensus recommendations of this multidisciplinary expert panel. Although they may serve as a set of general guidelines, each patient undergoing cTACE presents a unique combination of individual and tumor characteristics that must be carefully assessed and evaluated, and the best approach for any individual patient can only be decided by their treating interventional radiologist.

## 2 Patient selection

### Statement 2.1 (evidence level: 1, recommendation: A, agreement: 100%)

cTACE is recommended as a first-line treatment for unresectable, multifocal or large HCCs without vascular invasion or extrahepatic spread

cTACE can also be selectively performed in early-stage patients for whom radiofrequency ablation is unsuitable, due to either tumor location or medical comorbidities (Asian Pacific Association for the study of the liver).

**• Vascular invasion**

Patients with subsegmental (Vp1) or segmental (Vp2) levels of portal thrombosis may still be treated by cTACE.

**• Liver function**

Pretreatment Child-Pugh classification score A is recommended, but superselective embolization may still be considered in Child-Pugh B patients with hypervascular HCC.

At present, cTACE is primarily indicated in patients who are unable to receive curative treatment *via* surgery, liver transplantation or percutaneous ablation. Based on consensus recommendations on HCC treatment by the Asian Pacific Association for the Study of the Liver, cTACE is recommended as a first-line treatment for unresectable, large or multifocal HCCs without vascular invasion or extrahepatic spread. The prerequisite for any cTACE procedure is a comprehensive assessment of the patient’s clinical condition, with particular attention paid to the underlying liver disease and performance status (ECOG). Rather than considering the absolute tumor size in relation to the liver, an assessment should be made based on a patient’s pre-treatment liver function and the percentage of functional liver parenchyma that would remain after cTACE. In general terms, a pre-treatment Child-Pugh classification score of A is recommended, but superselective embolization may still be considered in patients with Child Pugh class B of score 7 (8). Several clinical trials and a recent meta-analysis have shown that, cTACE is capable of improving survival for patients with intermediate-stage HCC, with a median survival of 28.7 months (18) and 1- and 2-year survival of 57% and 31% (19), as opposed to 17.9 months (18) or 32% and 11% (19) for untreated patients.

cTACE can also be selectively performed in early-stage HCC cases for which RFA is unsuitable, due to either tumor location or medical comorbidities (23). If a local cure is achieved through an initial application of TACE, the method can be referred to as curative TACE (17).

However, the presence of portal vein tumor thrombosis (PVTT) is still often considered a contraindication for transplantation, curative resection and TACE (23–25). Studies have demonstrated that interruptions to the hepatic arterial supply in patients whose portal venous supply is already compromised can potentially lead to widespread segmental liver necrosis (25, 26). If PVTT is shown on pre-treatment imaging, we recommend following the grading scheme developed by the Liver Cancer Study Group of Japan (27), which has five grades in total (Vp0 to Vp4) which correspond to progressively more central thrombosis of the portal venous branches (27).

Nevertheless, there is evidence that certain patients with PVTT can still benefit from a modified form of cTACE, given good pre-treatment liver function and the existence of collateral blood flow around the site of portal vein obstruction (28, 29). Two recent studies have reported increased survival in HCC patients with PVTT after receiving cTACE. In the first, Luo et al. (10) found in their prospective nonrandomized study that median survival significantly improved in the cTACE group (n=84) as compared to the group that received conservative treatment (n=80), in either non-cirrhotic or Child A cirrhotic patients with PVTT. The authors reported median overall survival (OS), 1-year, and 2-year survival rates of 7.1 months, 30.9% and 9.2% respectively for the cTACE group, and 4.1 months, 3.8% and 0% for the conservative treatment group (p < 0.001) (10, 30). In the second study, Chung et al. (30) also reported significant improvements in the survival of HCC patients with Vp4 PVTT who were treated with cTACE (n = 83), as compared to supportive care alone (n = 42; median OS of 5.6 versus 2.2 months, p < 0.001).

While patients with Vp1 or Vp2 levels of portal thrombosis may still be treated by cTACE with modified methods as shown above, Vp3 patients have better received a combined regimen of both cTACE and radiotherapy, as some studies have shown an increase in patient survival using this method (31, 32).The presence of severe PVTT (Vp4) is, at present, a relatively stronger contraindication to TACE. In all cases with PVTT, other therapeutic modalities – such as Yttrium-90 (Y-90) (33), hepatic artery infusion chemotherapy (HAIC) (34), and immuno-target therapy (35) – should be considered in addition to cTACE.

In conclusion, HCC with portal or hepatic venous invasion should not be considered an absolute contraindication to cTACE.

## 3 Pre-treatment imaging

### Statement 3.1 (evidence level: 2, recommendation: A, agreement: 100%)

Dynamic multiphase CT and MRI are recommended as the first-line modalities for diagnosis and staging of HCC. In order to evaluate whether there are appropriate indications for transcatheter embolization of HCC and set up tailored treatment plans, and to allow proper assessment of the number, size and location of hepatic tumors, multiphasic computed tomography (CT) or dynamic contrast-enhanced magnetic resonance imaging (MRI) of the liver must be performed and must include at least three phases, including unenhanced, arterial, and portal venous phases. Suitable contrast injection rates and timing of each phase of scanning are essential to providing the highest possible HCC detection rate. Additional imaging modalities are required to evaluate the existence of extrahepatic disease, either within the abdomen or in distant organs (36). Previous studies have found differences in the detection rates of small HCCs with different imaging modalities, which may influence physician choice. The sensitivity of contrast enhanced ultrasound (US), CT and MRI for 1-2 cm HCC was found to be 26%, 44%, and 44%, respectively, with 100% specificity (37). However, liver-specific MRI contrast agents such as Gadoxetic acid (Gd-EOB-DTPA) have been shown to provide higher sensitivity and overall accuracy in HCC detection when compared to conventional multiphasic CT (38). Thus, gadoxetic acid-enhanced and diffusion-weighted MR imaging may be considered in cases with atypical enhancement patterns on multiphasic CT (39).

### Statement 3.2 (evidence level: 2, recommendation: B, agreement: 100%)

Late arterial phase is suggested for pretreatment imaging in the diagnosis of HCC

To improve the detection of hypervascular HCCs with multidetector computed tomography (MDCT), some investigators have previously recommended the use of double arterial phase imaging (40). However, Ichikawa et al. (2002) found no significant advantages when comparing double arterial phase imaging to single late arterial phase imaging for the detection of hypervascular HCC (41).

This panel recommends the use of MDCT with late arterial phase and portal phase imaging to optimize pre-treatment tumor detection. Late arterial phase images have been shown to maximize celiac axis attenuation, while portal venous phase images tend to demonstrate best attenuation of the portal vein and normal hepatic parenchyma. For hypovascular liver tumors, late arterial and portal venous phase images were shown to have superior tumor-to-parenchyma differences as compared to early arterial phase images. As for hypervascular tumors, late arterial phase images were also found to provide subjectively better tumor conspicuity compared to early arterial phase images (41–43).

## 4 Drug: lipiodol and chemo agents

### Statement 4.1 (evidence level: 5, recommendation: D, agreement: 88.4%)

Mixtures of anticancer drugs and Lipiodol should be based on the principle of water-in-oil emulsion, with relative proportions varying according to tumor size and vascularity.

Prior to injection, the anticancer drug(s) selected for cTACE are mixed with Lipiodol to form an emulsion. A W/O emulsion is favored when the volume of aqueous drug solution is lower than the volume of Lipiodol, and this should be maintained under most circumstances. The recommended ratio of drug to Lipiodol in Japan is typically 1:2, although this must be adjusted for factors such as tumor vascularity and size (15).The ratio of epirubicin to Lipiodol can also be used to adjust desired viscosity in the resultant W/O emulsion. The use of large-droplet W/O emulsions in intraarterial hepatic injection has also been theorized to limit the lung uptake while increasing the tumor uptake of iodized oil (44). Adequate mixing of an aqueous chemotherapy solution and Lipiodol requires at least 20 pumping exchanges through a 3-way stopcock, and the first push should be from the chemotherapy drug towards Lipiodol. The pumping speed strongly influences internal phase droplet size (and thus emulsion viscosity), with greater pumping resulting in smaller droplet sizes (45).

Our recommended method of preparing cTACE emulsion is *via* the 3-way stopcock method, using polycarbonate or glass syringes and preferably a metal stopcock that can resist degradation by the emulsion. During the mixing process, the chemotherapy drug should be pumped into the syringe containing Lipiodol in the first push, so as to favor a water-in-oil emulsion. A minimum of 20 alternating pumping exchanges through the stopcock are needed to ensure that the final emulsion contains the appropriate size of aqueous chemotherapy droplets (46). Lastly, it should be noted that the emulsion must be prepared immediately prior to intra-arterial administration, and it should be used directly after preparation. If some time has passed between injections during a single cTACE session, the mixture can be re-homogenized using the above-mentioned stopcock method.

### Statement 4.2 (evidence level: 1, recommendation: A, agreement: 100%)

- The most common single anticancer drugs are the anthracycline group agents, which include doxorubicin and epirubicin.
- The recommended dose of doxorubicin or epirubicin in a single session is 10-50 mg of body surface area based on tumor size.

A variety of cytotoxic anticancer drugs have been trialed for cTACE, and the most widely-used single-agent drugs in Taiwan are from the anthracycline group, which includes doxorubicin and epirubicin (47). According to European Association For The Study Of The Liver/European Organisation For Research And Treatment Of Cancer (EASL-EORTC) guidelines, there is at present insufficient evidence to recommend a single chemotherapeutical agent or combination of agents as being the best choice for cTACE (48). Nevertheless, a single-agent regimen involving either doxorubicin or cisplatin as a standard for fresh cases is recommended (49), with a common dosage of doxorubicin being approximately 10-50 mg of body surface area. The therapeutic effects can then be evaluated and a tailored re-treatment strategy formulated, with chemoembolization sessions scheduled 3–4 times per year during, which the anticancer agent may be changed or its dosage adjusted. It must be noted that there are currently no universally accepted criteria for determining the optimum dosage of chemotherapeutic agents in cTACE. Different authors have referenced various combinations of a patient’s body surface area, body weight, tumor burden or bilirubin level, and some have even use a fixed dose (50, 51). The dosing regimen reported also varies among different institutions in Taiwan, although the most common dose for doxorubicin tends to range 10-50 mg/m^2^ in a single session.

### Statement 4.3 (evidence level: 1, recommendation: A, agreement: no agreement)

- Several combination drug regimens have been reported, the most popular being cisplatin, doxorubicin, and mitomycin C
- Recommended dose: doxorubicin 50mg + mitomycin C 10mg

Although combined regimens involving multiple chemotherapeutic agents for cTACE have shown some promising results (52), there is currently a lack of adequate experience with their use in Taiwan. As such, this panel could not establish an agreement on which anticancer drugs to use in combination, and what the relative dosage of each drug should be. Nevertheless, the most popular combination regimen that may be considered includes cisplatin, doxorubicin, and mitomycin C.

## 5 Embolic agents: gelfoam, PVA, microsphere

### Statement 5.1 (evidence level: 3, recommendation: B, agreement: 94.2%)

• Both Gelatin sponge and non-resorbable calibrated microspheres can be used as embolic agents for cTACE, to be chosen based on the operator’s own judgement.

During each cTACE procedure, the infusion of a chemotherapeutic drug mixed with iodized oil is usually followed by the embolization of tumor-feeding arteries. This is done through the injection of gelatin sponge particles or other embolic agents, such as non-resorbable calibrated microspheres, and will significantly improve the cytotoxic effects of chemotherapy *via* additional ischemic effects. Gelatin particles, which may be purchased in pre-determined sizes or manually cut from larger pads, are the most commonly-used material for this purpose and have the main advantage of demonstrating complete vessel recanalization in 1–2 weeks after embolization (53). This provides temporary reduction in local blood flow to the tumor, which still allowing subsequent cTACE using the same tumor feeders. The size distribution of the gelatin sponge particles will differ according to the method used to make them. Reasonably uniform particle sizes are recommended and can be more consistently produced by cutting, rather than pumping (54).

The primary alternative to gelatin sponge particles is PVA microspheres with calibrated sizes of 100-300 μm. However, in patients with arterio-portal or arterio-venous shunt, larger particles (300-1000 μm) should be adopted. These come in two main types, one which is resorbable once infused into hepatic vessels and the other which is permanent. Since previous studies have not reported any additional benefit to using non-resorbable microspheres, use of the resorbable variant is recommended, in order to preserve the patency of tumor feeders and other arteries that may be valuable for subsequent cTACE treatment. To achieve optimal response in terms of tumor ischemia and shrinkage, as well as to ensure distal occlusion near the tumor vascular bed while preserving feeding segmental arteries, calibrated small microspheres are also recommended (55).Using microsphere sizes smaller than this increases the risk of shunting through hypervascular tumors and potential bile duct injury, while larger sizes may occlude feeding arteries without entering the vascular bed of the tumor (56).

## 6 Catheter positioning, embolization technique and end point

### Statement 6.1 (evidence level: 4, recommendation: C, agreement: 100%)

- A superselective (i.e., segmental or subsegmental) approach should be used whenever possible using a microcatheter.
- A lobar approach may be considered for large multinodular or bilobar tumors, depending on the patient’s condition.

Yamakado et al. reported that selective hepatic arterial embolization in

HCC patients can significantly improve survival (57). When performing a selective segmental or subsegmental cTACE, a microcatheter should be used whenever possible, and all tumor-feeding vessels must be precisely located and treated (58), especially subphrenic HCC nodules, which are usually supplied through the phrenic artery. Besides easing access to smaller vessels, deploying microcatheters also helps reduce the incidence of vasospasms during emulsion delivery and ensures the forward flow of embolic materials. To prevent refluxing to non-target vessels during infusion, the operator should take care that sufficient flow exists around the catheter to carry the drug/Lipiodol mixture or embolic material forward.

In patients for whom selective embolization is not feasible (for example, if the tumor is very large and multinodular or present in bilateral lobes), a lobar approach may become acceptable. If this is the case, the operator should maneuver the catheter into position in the left or right hepatic artery in such a manner that as many tumor feeding arteries as possible are covered, while avoiding vessels that supply extrahepatic organs.

### Statement 6.2 (evidence level: 4, recommendation: C, agreement: 100%)

Use of three-dimensional reconstructions obtained from CT or cone-beam CT is recommended, if available, to aid in the identification of tumor-feeding arteries.

“Targeted transarterial oily chemoembolization” is defined as superselective catheterization of a subsegmental or even more distal artery, followed by oily chemoembolization. The entire process is conducted with the assistance of a unified helical CT and real-time fluoroscopic system, usually referred to as C-arm cone-beam computed tomography (CBCT), which also allows intraoperative confirmation of embolization results *via* the CT component. Images produced by CBCT prior to superselective TACE can sometimes reveal subtle details of tumor-feeding arteries that may not be visible on digital subtraction angiography (DSA) alone. Thus, in addition to angiography of the hepatic and mesenteric artery, which is routinely obtained to identify liver arterial anatomy, major tumor feeders, and obvious portal or venous shunts, rotational angiography with C-arm CBCT may also be used to delineate tumor arterial supply with greater assurance. Additionally, the multiplanar reconstructions obtained during rotational imaging can be used to guide catheter tip positioning (59, 60).

By repeating selective arteriography with follow-up CBCT scans during catheter advancement through a segmental or subsegmental artery, a precisely targeted embolization of very distal tumor feeding branches may be achieved. This has the major advantage of strengthening the effect of chemoembolization on a target lesion while reducing damage done to surrounding healthy liver parenchyma. A reduction of up to two-thirds of the amount of anticancer agent and iodized oil used may be realized with this technique, while the need for gelatin sponge particles is also drastically reduced. Another merit of using a C-arm CBCT system, as mentioned above, is the ability to immediately confirm with CT images upon completion of cTACE whether targeted lesions were completely treated (61).

### Statement 6.3 (evidence level: 3, recommendation: B, agreement: 100%)

Complete stasis up to the catheter tip when placed in subsegmental arteries.

• Lipiodol visualization in distal portal vein radicals of embolized areas, and completely occlusion of tumor feeding branch after gelatin sponge injection

The degree of portal vein visualization due to overflow of embolic materials has been divided into three grades by Miyayama et al. (62), as follows: Grade 0 = no obvious visualization of the portal vein or its branches (no visualization); Grade 1 = visualization of the portal vein adjacent to the tumor (slight visualization); and Grade 2 = marked visualization of the portal veins in the whole embolized area or extending into surrounding non-embolized areas (marked visualization). The optimal endpoint of a cTACE procedure can be recognized by portal vein visualization in the entire embolized area, along with complete blockage of tumor-feeding branch(es). Iodized oil injection should cease when portal veins extending to embolized area become visible, while the injection of gelatin sponge particles is deemed complete when tumor feeding branch(es) have been completely obstructed. So long as excessive overflow does not occur, the catheter tip should be kept in a subsegmental artery while injecting iodized oil, until complete vessel stasis is achieved. Injection of a small amount of gelatin sponge particles can subsequently be performed in the same location to complete the cTACE session (63).

### Statement 6.4 (evidence level: 5, recommendation: C, agreement: 100%)

To identify residual tumors, post-embolization angiography or CBCT images may be obtained immediately after completion of a cTACE session.

The radiopaque nature of Lipiodol allows an operator to continuously visualize the flow of chemotherapeutic agents during infusion, which increases the safety and efficacy of cTACE. The Lipiodol-drug emulsion should be monitored in real-time *via* fluoroscopy until the vascular bed of the target tumor is completely saturated and stasis in peripheral branches next to the vascular bed is observed. Tumors with more complete Lipiodol opacification correlate with better treatment response, including lower rates of local recurrence (64) and higher rates of complete tumor necrosis (62). Therefore, we recommend repeating CBCT or angiography immediately after Lipiodol delivery in order to confirm tumor saturation and the occlusion of feeding vessels (59).

### Statement 6.5 (evidence level: 2-3, recommendation: B, agreement: 100%)

#### 6.5.1 Substasis angiographic endpoints during cTACE

##### 6.5.1.1 Subjective angiographic chemoembolization endpoint scale levels II and III

#### 6.5.2 Preservation of flow in the major lobar and segmental arteries when performing less-selective cTACE

##### 6.5.2.1 A ‘‘tree-in-the winter’’ appearance

##### 6.5.2.2 Occlusion of small tumor-feeding radicals

There is at present no global consensus on an optimal embolization endpoint for TACE (65), resulting in great variability between individual operators and institutions. However, the two primary goals of embolization are as follows: first, to induce ischemia and necrosis in the target tumor by reducing blood flow to the lesion, and second, to enhance the effects of administered cytotoxic agents (66, 67). An appropriate amount of embolization that is tailored to each target lesion is crucial, since both under- and over-embolization during cTACE can lead to undesirable effects; inadequate embolization can obviously result in poor treatment response (68, 69) and activate angiogenic growth factors that increase HCC progression (70–72), while over-embolization may increase liver toxicity (73). In addition, excessive embolization during cTACE may permanently block a tumor’s feeding vessels, hindering future cTACE procedures and potentially affecting patient survival. Patients with Child-Pugh class B or C disease require extra care during embolization, since baseline liver dysfunction tends to increase the risk of post-TACE liver failure (74).This is a wide-spread issue in HCC patients, since up to 80% have been found in autopsy to have underlying cirrhosis (75).

As the aforementioned findings show, a more consistent set of criteria for determining appropriate embolization endpoints is urgently needed, and some researchers have attempted to establish such a system. Jin et al. introduced the SACE scale to describe angiographic endpoints of cTACE (**Table 1**), which this panel found to be quite useful (65). Level I represents under-embolization, which is shown as minimal or no change in tumor blush or antegrade flow in feeding vessels on DSA images after cTACE. Level IV represents over-embolization, which is defined as the elimination of both tumor blush and antegrade blood flow after embolization. Jin et al. also demonstrated that embolization to an intermediate, sub-stasis endpoint (SACE levels II and III) during cTACE improves survival, when compared with embolization to a more complete stasis endpoint (level IV). Since the optimal end-point of embolization is the subtotal stasis of tumor blood flow, the interventional radiologist should aim to achieve a “tree-in-the winter’’ fluoroscopic appearance. This entails the occlusion of terminal tumor-feeding branches while preserving flow in the lobar and segmental hepatic arteries, which will allow repeat treatment through these vessels in the future (76, 77). Stopping embolization at SACE II-III (substasis) is especially important in patients with increased bilirubin levels and/or those with high likelihood to undergo repeated cTACE, both to preserve liver function and to facilitate future treatment. Complete stasis (SACE IV) may be relatively more acceptable in cases with normal bilirubin levels and/or a low likelihood to be retreated in the same targeted artery. In general, however, interventional radiologists should aim for intermediate, sub-stasis angiographic endpoints of cTACE for most patients.

**Table 1:** Subjective angiographic chemoembolization endpoint scale.

Level	residual antegrade flow	residual tumor blush
I	normal	normal
II	reduced	reduced
III	reduced	none
IV	none	none

## 7 Treatment schedule

### Statement 7.1 (evidence level: 1, recommendation: A, agreement: 88.4%)

- On-demand cTACE procedures up to 3 or 4 times per year according to treatment response.
- At least two cTACE procedures should be performed before treatment is abandoned due to lack of response.
- Patients with types of HCC unsuitable for cTACE – including confluent multinodular type, massive or infiltrative type, poorly differentiated type, and intrahepatic multiple disseminated nodules and nodules that exceed the up-to-7 criteria – should first seek out systemic therapy (e.g., immuno-target therapy) and/or locoregional therapy (e.g., y-90 radioembolization and HAIC).

#### 7.1.1 cTACE failure/refractoriness

We recommend that a minimum of two sequential cTACE procedures be performed in a treatment cycle, since a single chemoembolization may be insufficient to effectively treat intermediate stage HCC. While repeating cTACE is widely known to prolong overall patient survival (78), current guidelines do not specify the conditions and timing under which repeat treatment should be performed. In the opinion of this expert panel, ‘on-demand’ embolization (i.e. adjusting the number of treatment sessions based on tumor response) up to 3 or 4 times per year provides the most effective and flexible treatment option, while maintaining the patient’s quality of life (79–81). According to the “Management consensus guideline for HCC: 2016 updated by the Taiwan liver cancer association and the gastroenterological society of Taiwan”, TACE-refractory HCC should be considered when evidence of disease progression or viable components making up over 50% of a tumor are observed on CT or MR images, for target lesions which have been adequately treated in two or more sessions within a 6-month period (82). For refractory HCC cases, alternative therapeutic options should be sought in the absence of response after two cTACE sessions (83).

#### 7.1.2 cTACE-unsuitability

Some clinical conditions may prevent HCC patients from experiencing survival benefits from cTACE (22), including (I) the unlikeliness to respond to cTACE due to confluent multinodular type, massive or infiltrative type, poorly differentiated type, or intrahepatic multiple disseminated nodules and (II) the likeliness to develop TACE failure/refractoriness and/or deterioration of liver function in nodules exceeding the up-to-7 criteria (84). Because survival does not differ between non-responders to cTACE and untreated patients, cTACE is not recommended to be repeated in cases in which OR was not achieved by prior cTACE (85), since repetition may lead to sarcomatous or biliary-mixed type changes and the development of an aggressive type of cancer that does not respond to cTACE (86, 87). Furthermore, repeated cTACE procedures might impair liver function, with the incidence higher in patients exceeding the up-to-7 criteria than in those within them (88).

Radiological assessment of tumor response and changes in Child-Pugh score are two common patient prognostic factors after cTACE, while Golfieri et al. proposed that tumor size and radiologic response be taken into account when considering further treatment (89).

In the current era of multi-molecular targeted agents and immune checkpoint inhibitors, lenvatinib has been the only first-line agent to demonstrate an OS benefit over cTACE (37.9 vs. 21.3 months) in TACE-naïve patients with tumor burdens exceeding the up-to-7 criteria (90). Approximately 70% of such patients have undergone cTACE after initial lenvatinib therapy; thereafter, initial lenvatinib therapy with subsequent selective cTACE has been proposed for intermediate-stage HCC with high tumor burden (91). Noting that the above conclusions come from a single retrospective propensity-score matched study (90), further prospective randomized control trial is warranted to confirm the effectiveness of this treatment strategy (91). In the 2019 APPLE consensus document (22), country-specific issues such as healthcare insurance or approval status were not considered, so it is necessary to make adjustments appropriate to each situation, especially to account for the highly-variable costs of medical treatments in each country.

## 8 Post-treatment care and imaging follow-up

### Statement 8.1 (evidence level: 3, recommendation: B, agreement: 100%)

- Post-embolization syndrome consisting of fever, abdominal pain and increased serum white blood cell count is an expected side-effect of cTACE and should not be regarded as a complication.
- Pain relievers, prophylactic antibiotics, antiemetic therapy, and gastric protection should be provided according to standard institutional protocols
- Both the non-enhancing and Lipiodol retention areas should be considered necrotic before treatment is abandoned due to lack of response

Post-chemoembolization syndrome, which occurs in approximately 60% to 80% of patients who undergo cTACE, is typically associated with elevation of hepatic transaminases, transient abdominal pain, and fever. Routine monitoring of vital signs and conservative treatment are all that are required in most patients, as it is self-limiting and resolves itself within 3–4 days. Post-embolization hospitalization may be prolonged, however, in patients who develop these symptoms. Even though there is currently no solid evidence supporting the routine use of antibiotic prophylaxis, many physicians still recommend antibiotic treatment for 3–7 days after cTACE treatment, using appropriate agents that cover Gram-negative enteric pathogens (92, 93).

### Statement 8.2 (evidence level: 4, recommendation: C, agreement: 100%)

Major complications such as liver infarction, biloma, surgical cholecystitis, gastrointestinal ulceration or hemorrhage, vascular dissection occur in less than 1% of procedures, while the incidence of liver failure and patient mortality in 30 days is less than 4%.

Complications may occur in an estimated 10% of cTACE patients. Severe adverse events, which include liver infarction, biloma, surgical cholecystitis, gastrointestinal ulceration or hemorrhage, and vascular dissection, each have an incidence rate of < 1%. Meanwhile, the expected rates of liver failure and patient death within 30 days post-TACE are 2.3% and 2-4%, respectively (94). Liver insufficiency leading to hepatic failure is the most common cause of death, although the overall mortality rate remains low at approximately 0.6% (20). This suggests that an accurate assessment of a patient’s baseline liver function parameters is the most important factor in lowering the risk of treatment-related mortality, as well as reducing overall liver-related adverse effects.

### Statement 8.3 (evidence level: 5, recommendation: C, agreement: 100%)

- To evaluate tumor response and plan further therapy, serum alpha-fetoprotein (AFP) level and follow-up dynamic liver CT/MRI should be examined 8-10 weeks after a course of cTACE.
- If treatment of both lobes of the liver is planned, additional imaging between sessions may be performed based on operator preference.

After all hepatic tumors have been treated, follow-up dynamic liver CT or MRI should be arranged within 8-10 weeks. Additional imaging of the liver may also be considered if bilobar embolization is being planned. When imaging is unable to confirm or rule out the presence of residual tumors or necrotic/fibrotic tumor remnants, serum AFP or PIVKA II may be helpful in measuring the degree of treatment response (95).

### Statement 8.4 (evidence level: 1, recommendation: A, agreement: 100%)

- The modified Response Evaluation Criteria in Solid Tumors (mRECIST) guideline is recommended for classification of HCC response to cTACE.
- For patients with no evidence of residual viable disease, imaging follow-up is recommended every 3 months.

The mRECIST criteria rely on assessments of tumor devascularization to classify treatment responses (96, 97), and have proven to be the most accurate system for evaluating cTACE in terms of predicting time to disease progression and overall patient survival (98). For patients without active disease upon initial follow-up after embolization, routine liver imaging should be arranged every 3 months.

### Statement 8.5 (evidence level: 4, recommendation: B, agreement: 100%)

- When CT is used for disease evaluation, both non-enhancing areas and areas with iodized oil retention should be considered part of tumor necrosis.
- Lipiodol retention in treated lesions may cause difficulties in assessing residual or recurrent tumor using contrast-enhanced CT. In such cases, contrast-enhanced liver MRI may be chosen as an alternative modality.

Takayasu et al. demonstrated that CT is suitable for evaluating the efficacy of cTACE for HCC, based on the assumption that the portions of the tumor which retain iodized oil are necrotic. The authors showed that tumor size reduction measured on CT does not directly correlate with the therapeutic effect of chemoembolization, and that we should instead focus on the extent of tumor necrosis (99). Signs of tumor necrosis include Lipiodol uptake and the absence of arterial-phase enhancement in areas where it was present prior to chemoembolization. In certain situations, the high density of retained Lipiodol in already-treated lesions may mask the presence of residual or recurrent tumors in its vicinity when using contrast-enhanced CT. Dynamic contrast-enhanced MRI may be considered an alternative follow-up modality in these cases, since it is much less affected by Lipiodol-induced artifacts. In MR imaging, absent arterial enhancement in the areas of the tumor where it was present prior to therapy is the principal determinant of tumor necrosis (100).

Note:

Some controversies regarding the role of Lipiodol in cTACE exist in the literature. Before objectively discussing these controversies, we should establish a clear and precise nomenclature, as transarterial embolization terms have been inconsistently used across a multitude of studies:

1. Lp-TACE: lipiodol + anticancer agent + embolizer (gelfoam/PVA)
–Lp-TACE: anticancer agent + embolizer (gelfoam/PVA)
3. Lp-TAE: lipiodol + embolizer (gelfoam/PVA)
–Lp-TAE: embolizer alone (gelfoam/PVA)

Although lipiodol is widely adopted in TACE protocols, its exact role as a carrier vehicle, microembolizer, and/or both is still disputed. As a carrier, emulsifications of lipiodol and drugs are unstable, and the two components begin to separate as soon as they are injected into the hepatic arterial circulation (101, 102). Numerous studies have shown no differences between the injection of chemoagents as an intra-arterial infusion or as part of conventional TACE protocols, in terms of pharmacokinetic parameters or toxicity (103–107). Kobayashi et al. reported that there were no differences in plasma epirubicin concentration after injection (72) of whatever oil-in-water, water-in-oil, or suspension form of epirubicin-lipiodol emulsion. To date, there is also no evidence of the superiority of any chemotherapeutic agent, either alone or of monotherapy, versus combination therapy^48.^

3 RCTs compared the therapeutic effect of TACE versus TAE, with two trials favoring TAE (108, 109), and one favoring TACE (110). The 1- and 2-year survival rates in the two RCTs favoring TAE (Lp-TAE) were 74.4 & 51.3% vs 65.1 & 42.4%, *P*=.209 (108) and 72.5 & 39.5% vs 52.5 & 26.2% (*P*>.05) (109), while the median survivals were 25.3 months (-Lp-TAE) and 28.7 months (TACE), favoring the TACE trial (*P*>.05) (110). 2 non-randomized studies also reported that TACE had significantly superior survival than TAE, with 1- and 2-year survivals of 65% & 39% vs 39% & 13%, & 73% and 31% vs 39% & 10%, respectively (18, 111). However, in the latter three studies, TAE were all performed with gelfoam fragments only, without associated lipiodol injections (-LpTAE) (18, 110, 111). Moreover, Pelletier et al. and Bruix et al. both conducted RCT studies and found there was no survival benefit to treating unresectable HCC without lipiodol, by either chemoembolization with doxorubicin and gelfoam powder (-Lp-TACE) or gelfoam alone (-Lp-TAE) when compared to symptomatic treatment, with survival rates of 24% versus 31% at 12 months (*P*>0.05) in the former and 49% versus 50% at 24 months (*P*=.072) in the latter (112, 113).

From a large number of both randomized or nonrandomized studies and a systematic review in 2016 conducted by Lenioni et al, it is reasonable to conclude that “the key component of TACE is lipiodol … which is used both as a vehicle to carry and localize the chemotherapeutic agent inside the tumor and as a microembolic agent for tiny tumor vessels” (20). And the mechanism of TACE or TAE can be summarized as: 1) for TACE, the *choice* of bolus anticancer agents infusion play little role in the HCC treatment, insofar as no particular chemoagents have been proven to be more HCC-sensitive than any others and because of the early separation of the chemoagent from the emulsion, no matter the preparation; 2) small-droplet lipiodol oil act as an embolizer for tumor microvasculature per se, embolizing the distal hepatic arterioles and overflowing to the portal venules, thus playing the key role in TACE and in TAE, especially for small HCC less than 4-5 cm; 3) gelfoam particles of 1-2 mm act as an adjunct embolizer to occlude (sub)segmental hepatic arteries, delaying lipiodol washout in the subsegmental hepatic arteries and providing adequate lipiodol-retained duration for irreversible tumor cell ischemia and death.

## Author contributions

Conceptualization: P-YC, R-CL, P-CL, Y-SL, VC, D-KW, Y-FC, J-IH, H-ST, C-HW, C-FH, R-HW, M-CC, H-MC, S-MC, C-LC and H-LL. Writing – original draft preparation: P-YC. Writing – review and editing: H-LL. Supervision: R-CL, P-CL, Y-SL, VC, D-KW, Y-FC, J-IH, H-ST, C-HW, C-FH, R-HW, M-CC, H-MC, S-MC, and C-LC. All authors contributed to the article and approved the submitted version.
